# Optimization based trajectory planning for real-time 6DoF robotic patient motion compensation systems

**DOI:** 10.1371/journal.pone.0210385

**Published:** 2019-01-11

**Authors:** Xinmin Liu, Rodney D. Wiersma

**Affiliations:** Department of Radiation and Cellular Oncology, The University of Chicago, Chicago, IL 60637, United States of America; North Shore Long Island Jewish Health System, UNITED STATES

## Abstract

**Purpose:**

Robotic stabilization of a therapeutic radiation beam with respect to a dynamically moving tumor target can be accomplished either by moving the radiation source, the patient, or both. As the treatment beam is on during this process, the primary goal is to minimize exposure of normal tissue to radiation as much as possible when moving the target back to the desired position. Due to the complex mechanical structure of 6 degree-of-freedom (6DoF) robots, it is not intuitive as to what 6 dimensional (6D) correction trajectory is optimal in achieving such a goal. With proportional-integrative-derivative (PID) and other controls, the potential exists that the controller may generate a trajectory that is highly curved, slow, or suboptimal in that it leads to unnecessary exposure of healthy tissue to radiation. This work investigates a novel feedback planning method that takes into account a robot’s mechanical joint structure, patient safety tolerances, and other system constraints, and performs real-time optimization to search the entire 6D trajectory space in each time cycle so it can respond with an optimal 6D correction trajectory.

**Methods:**

Computer simulations were created for two 6DoF robotic patient support systems: a Stewart-Gough platform for moving a patient’s head in frameless maskless stereotactic radiosurgery, and a linear accelerator treatment table for moving a patient in prostate cancer radiation therapy. Motion planning was formulated as an optimization problem and solved at real-time speeds using the L-BFGS algorithm. Three planning methods were investigated, moving the platform as fast as possible (platform-D), moving the target along a straight-line (target-S), and moving the target based on the fastest descent of position error (target-D). Both synthetic motion and prior recorded human motion were used as input data and output results were analyzed.

**Results:**

For randomly generated 6D step-like and sinusoidal synthetic input motion, target-D planning demonstrated the smallest net trajectory error in all cases. On average, optimal planning was found to have a 45% smaller target trajectory error than platform-D control, and a 44% smaller target trajectory error than target-S planning. For patient head motion compensation, only target-D planning was able to maintain a ≤0.5mm and ≤0.5deg clinical tolerance objective for 100% of the treatment time. For prostate motion, both target-S planning and target-D planning outperformed platform-D control.

**Conclusions:**

A general 6D target trajectory optimization framework for robotic patient motion compensation systems was investigated. The method was found to be flexible as it allows control over various performance requirements such as mechanical limits, velocities, acceleration, or other system control objectives.

## Introduction

Modern radiation therapy (RT) delivery methods have evolved to the extent that technologies such as Intensity Modulated Radiation Therapy (IMRT) or Volumetric Modulated Arc Therapy (VMAT) can now tightly conform radiation to the 3D shape of a target with millimeter (mm) accuracy [1, 2]. This opens up the possibility of further radiation dose escalation to the tumor while still keeping doses low to surrounding organs-at-risk (OAR). However, as RT becomes increasingly conformal, and tends toward higher dose over fewer fractions, the issue of patient motion becomes ever more critical to address [3]. In stereotactic radiosugery (SRS) or stereotactic body radiation therapy (SBRT) small, highly focused, and accurate radiation beams are used to escalated doses in 1-5 treatments as opposed to smaller doses over 20-30 treatments. In such cases motion-related errors of even 1-2 mm can be significant.

Adaptive radiation therapy (ART) methods aim to address the RT motion management problem by using image guidance to locate the position of targets and then adapting the radiation dose to conform to these new positions. One form of ART is where dose re-planning is rapidly performed before the start of each treatment fraction while the patient is on the linear accelerator (LINAC). A daily cone-beam CT (CBCT) is taken by the LINAC, and deformable image registration (DIR) and dose recalculation is performed to correct the tumor and OAR volumes for shrinkage, expansion, motion, or other positional changes that may have occurred after the initial planning CT scan [4]. A number of studies have evaluated the benefits of ART, indicating improved target coverage and reduced normal tissue toxicity [5–8]. However, current ART modalities cannot address changes that may take place during radiation beam delivery. Lung, prostate, pancreas, liver, and other thoracic and abdominal tumors have been shown to move as much as 35 mm with breathing, rectal filling, intestinal gas, or other types of biological motion [9, 10]. Clinical methods to manage such motion include beam gating, abdominal compression, or breath-hold [11–14]. In recent years, significant research has been invested in exploring dynamic motion management strategies, such as moving the radiation source, the patient, or both [15, 16]. Here the primary goal is to stabilize the radiation beam with respect to a dynamically moving target. As the target can move along both translational (x, y, z) and rotational (pitch, row, yaw) axes as a function of time, precise positioning of the patient and LINAC becomes extremely challenging. This is especially true for single-isocenter-multiple-target SRS, where small rotations can lead to large positional errors for targets located away from isocenter, resulting in poor or missed target dose coverage and degraded treatment effectiveness [17–22].

To effectively deal with intra-fractional patient motion, it is necessary to use a motion control algorithm that can respond to both translational and rotational deviations in real-time. Various motion control algorithms have been investigated in RT, with the most focusing on real-time lung tumor motion compensation using a moving patient treatment table. D’Souza et al. employed a 1D controller that was used to drive a LINAC table such that it moves in phase with target, but in the opposite direction, to maintain the patient translational position [15]. Other methods used adaptive filter prediction and predetermined dynamic models to determine appropriate positioning commands [23, 24]. A proportional integral (PI) controller was developed and evaluated for a 1 degrees-of-freedom (DoF) treatment table tracking system to counter steer respiratory tumor motion [25]. A coordinated dynamics-based proportional integral derivative (PID) control strategy was used to control the robotic system for continuously tracking translational position of the tumor [26]. The efficacy of this method was investigated by extensive computer simulation on two commercially available couches. Use of a linear Kalman filter to predict the surrogate motion with a linearized state space model to predict table position and velocity were investigated [27]. A model predictive control (MPC) of a robotic treatment table for motion compensation was reported in [28]. To maintain dynamic behavior of systems in the face of perturbations and other uncertainties, a robust control for parallel robotic platforms was investigated and compared with a widely used PID approach using extensive computer simulations [29]. For robotic SRS based head motion with angular stabilization, a decoupling control method for a 4D (xyz+pitch) robot was investigated [30, 31].

As the radiation beam is on during real-time patient 6DoF motion compensation, the target correction trajectory must be optimal both spatially and temporally in order to reduce unnecessary exposure of healthy tissue to radiation. Most prior investigations were limited to only 3DoF translational (xyz) motion, and therefore are not suitable to 6DoF robotic systems. Applying such methods to a 6DoF robot can result in a correction that may reach the desired position following a path that is slow, highly curved, or otherwise suboptimal. Additionally, most prior works have focused on respiratory motion compensation, and have employed the use of prediction algorithms to buffer against system lag times. Such methods are therefore not suitable for prostate, liver, head, or other types of motion that can be highly unpredictable [32]. To address these issues, we have developed for the first time, a novel optimization based motion controller that takes into account both robot mechanical constraints and patient safety tolerances, and performs a search over all possible 6D correction trajectories in order to select one that is most optimal. The method is universal as it can be applied to any robotic system provided that the robot’s joint configuration is well-known. Since the method formulates the correction trajectory problem as an objective function to be optimized, various constraints can be easily applied on actuator mechanical limits, patient velocities, and other aspects of the system that must operate within fixed limits during the motion compensation process. We demonstrate that standard convex optimization methods can be used to solve this problem at real-time speeds. As the controller responds with the most optimal path, robot lag time is also reduced, allowing for motion compensation in cases of slow unpredictable motion without the use of prediction. In cases of rapid, but predictable motion, such as lung based tumors or other respiratory coupled targets, the lower lag time reduces the prediction window duration. This is beneficial from a patient safety standpoint, where prediction accuracy deteriorates the further an algorithm must predict in the future.

## Materials and methods

### Robotic system simulations

**Fig 1:**
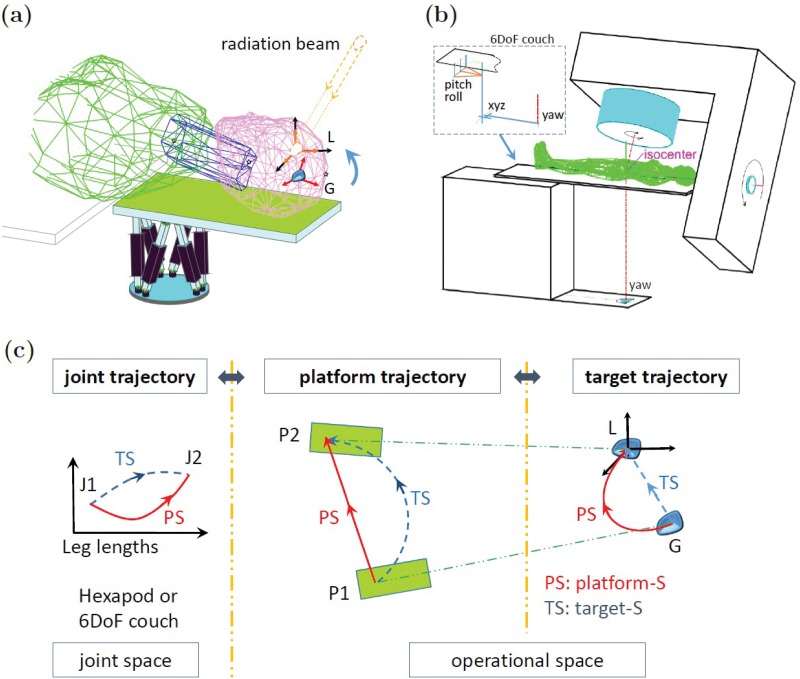
Illustrative diagram depicting 6DoF medical robotic systems (a)(b) and the concept of motion trajectories in different spaces (c). Three types of trajectories involved in the motion compensation: joint, platform, and target. To send the target from *G* back to the desired position *L* in operational space, a motion of the platform from *P*1 to *P*2 is needed, and such a motion can be implemented by changing the actuator lengths from *J*1 to *J*2 in the joint space. As shown, performing a straight-line trajectory in one space can result in a highly curved trajectories in other spaces.

Similar to other works that have investigated robotic motion control, we employ the use of computer simulation to test various kinematic and dynamic properties of the robotic system [26, 27]. As shown in Fig 1, dynamic computer simulations of a 6DoF SRS system and a 6DoF LINAC treatment table were constructed using Matlab (Mathworks, Natick, MA). The SRS simulation consists of six linear actuators arranged in a parallel kinematic fashion that performs real-time head motion stabilization by moving a platform (end-effector) that supports the patient’s head [33, 34]. The treatment table simulation is modeled after a commercial product (PerfectPitch, Varian Medical System, CA), and is a serial-parallel kinematic system where the platform moves the entire patient.

The clinical target to be stabilized with respect to the treatment beam will be considered as a rigid body and thereby represented by 6D coordinates that include the linear position (x, y, z) and the orientation (pitch, roll, yaw). The target is located at position *G* in the LINAC coordinate frame, and is tracked in real-time during the treatment using a suitable 6DoF patient motion monitoring device such as kV fluoroscopy [35, 36], infrared (IR) markers [32], or 3D surface imaging [37]. The desired position (setpoint) is given as *L*, and corresponds to the LINAC isocenter. As shown in Fig 1, the motion control problem can be divided into two spaces: joint (robot actuators) and operational (platform and target). In the simplest case, where a robot’s axes are all aligned with the LINAC frame, and one only considers 3D translational motion (xyz), the joint, platform, and target will all move along the same trajectory. In this case one does not require trajectory planning, as a straight-line trajectory from *G* to *L* will always be optimal. However, with a 6DoF robot, that is capable of both translational and rotational motion, the joint, operational, and target trajectories may differ substantially. For example, a straight-line target trajectory (blue dashed line), may result in highly curved joint and platform trajectories. It is no longer intuitive as to what 6D trajectory is now optimal both spatially and temporally.

### Robotic motion compensation scheme

**Fig 2:**
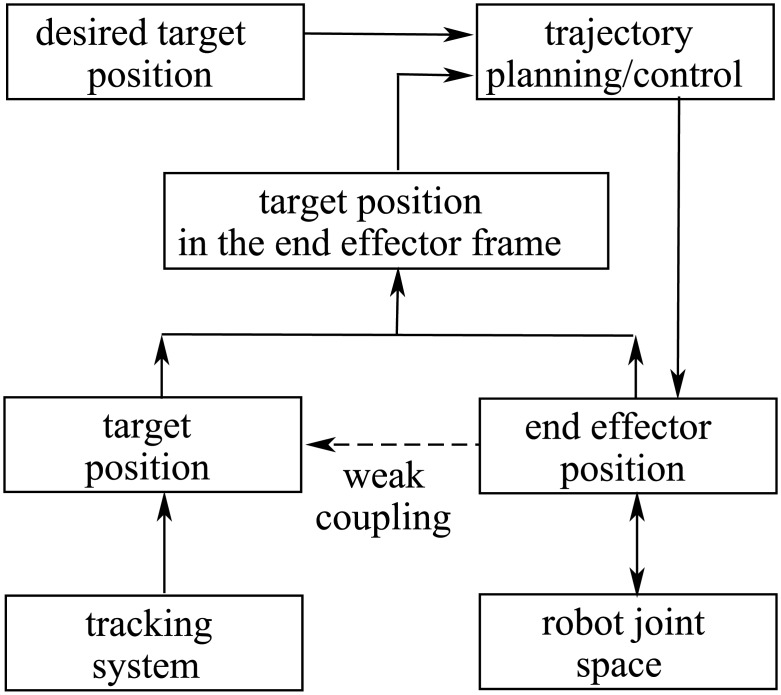
Signal flow diagram of the robotic motion compensation system.

For a given measured target position with a displacement away from the desired setpoint, the first step is to compute what is the required robot platform position for returning the target back to the setpoint. This can be done by the signal flow diagram in Fig 2. Suppose the measured target position is (*r*_*g*_, *ψ*_*g*_), the position of the platform is (*r*_*u*_, *ψ*_*u*_), and the position of the target in the platform frame is (*r*_*gu*_, *ψ*_*gu*_). Then,

$$\begin{array}{c} r_{g}=r_{u}+\Omega {\left(\psi_{u}\right)}^{\text{T}}r_{gu},\;\Omega \left(\psi_{g}\right)=\Omega \left(\psi_{gu}\right)\Omega \left(\psi_{u}\right), \end{array}$$ (1)

where Ω(⋅) is the direction cosine matrix, and (*r*_*gu*_, *ψ*_*gu*_) is the target position in the platform coordinate frame. The position (*r*_*gu*_, *ψ*_*gu*_) can be calculated by using the current platform position and the measured target position,

$$\begin{array}{c} r_{gu}=\Omega \left(\psi_{u}\right)\left(r_{g}-r_{u}\right),\;\Omega \left(\psi_{gu}\right)=\Omega \left(\psi_{g}\right)\Omega {\left(\psi_{u}\right)}^{\text{T}}. \end{array}$$ (2)

Assume that the target position with respect to the platform remains unchanged during each robot time cycle, it can be verified that by moving the platform to the following position $\left({\hat{r}}_{u},{\hat{\psi }}_{u}\right)$,

$$\begin{array}{c} {\hat{r}}_{u}={\hat{r}}_{g}-\Omega {\left({\hat{\psi }}_{u}\right)}^{\text{T}}r_{gu},\;\Omega \left({\hat{\psi }}_{u}\right)=\Omega {\left(\psi_{gu}\right)}^{\text{T}}\Omega \left({\hat{\psi }}_{g}\right), \end{array}$$ (3)

the target will move back to the desired position.

The actuator length of the robot can be calculated accordingly based on the platform position (*r*_*u*_, *ψ*_*u*_) by inverse kinematics. Suppose the coordinates of the robot joints in the LINAC coordinates frame is $D_{wi}\in {R\;\;}^{3}$, and the coordinates of the corresponding joints at in the platform frame is $D_{ui}\in {R\;\;}^{3}$. Then the actuator length is given by

$$\begin{array}{c} \ell_{i}=∥r_{u}+\Omega {\left(\psi_{u}\right)}^{⊤}D_{ui}-D_{wi}∥, \end{array}$$ (4)

for *i* = 1, 2, ⋯, 6.

For easy reference, let us make the following denotations. Denote the 6D target and platform position vectors as *x* ≔ (*r*_*g*_; *ψ*_*g*_) and *u* ≔ (*r*_*u*_; *ψ*_*u*_), respectively. Here (*y*; *z*) is used to denote the column vector (*y*^⊤^ *z*^⊤^)^⊤^. Denote initial positions as *x*_*o*_ = *x*(0) and *u*_*o*_ = *u*(0), initial actuator length as 6D vector *ℓ*_*o*_ = *ℓ*(0). Denote the desired target position as $\hat{x}≔\left({\hat{r}}_{g};{\hat{\psi }}_{g}\right)$, the required position of platform that pushes the target back to the desired position as $\hat{u}≔\left({\hat{r}}_{u};{\hat{\psi }}_{u}\right)$, and the corresponding actuator length as $\hat{\ell }$.

### Optimization based trajectory planning

Once the start and desired positions of the platform are known, the next step is to design a suitable trajectory between these two points. For reference, a path denotes the locus of points in a space and is a pure geometric description of motion, while a trajectory is a path with timing information [38]. For robotic motion compensation it is critical that the target trajectory converges to the desired position in an optimal way, such that a minimum time, shortest path, or steepest descent of positional error is achieved. Failure to do so will lead to unnecessary exposure of healthy tissue to radiation and poor tumor dose conformality.

To solve this problem, trajectory planning will be considered by three trajectories in two spaces: joint trajectory in the joint space, and platform trajectory and target trajectory in the operational space (Fig 1). When the platform moves along a trajectory in the operational space, *u*(*k*), *k* = 0, 1, ⋯, *n* with *u*(0) = *u*_*o*_ and $u\left(n\right)=\hat{u}$ for certain *n*, the target will approach the desired position following a certain trajectory in the operational space, *x*(*k*), *k* = 0, 1, ⋯, *n* with *x*(0) = *x*_*o*_ and $x\left(n\right)=\hat{x}$. To implement such an platform trajectory, the actuator length should thus follow a certain trajectory in the joint space, *ℓ*(*k*), *k* = 0, 1, ⋯, *n* with *ℓ*(0) = *ℓ*_*o*_ and $\ell \left(n\right)=\hat{\ell }$.

Due to the many parameters involved, it is not intuitive as to what trajectory is optimal both spatially and temporally. As can be seen in Fig 1, following the shortest platform path (platform-S) is not necessarily the best path for the target. Fortunately, such problems can be solved efficiently in real-time by use of optimization algorithms such as the quasi-Newtonian Limited-Broyden-Fletcher-Goldfarb-Shannon (L-BFGS) algorithm [39], Proximal Operator Graph Solver (POGS) [40–43], or other fast optimization algorithms. As a rule, the correction trajectory must meet control objectives and are subject to the robot’s mechanical constraints and system’s dynamic constraints. The robot’s actuator constraints can be specified in joint space as,

$$\begin{array}{c} -\Delta \ell_{\text{max}}⪯\ell \left(k+1\right)-\ell \left(k\right)⪯\Delta \ell_{\text{max}}, \end{array}$$ (5)

where Δ*ℓ*_max_ is the limit of actuator length change in each step. And the dynamic constraints can be specified in operational space as

$$\begin{array}{c} -v_{\text{max}}⪯u\left(k+1\right)-u\left(k\right)⪯v_{\text{max}}, \end{array}$$ (6)

where the vector *v*_max_ is the maximum 6DoF motion of the platform in one step.

By use of (4), the constraint (5) can be represent as the following platform motion constraints,

$$\begin{array}{c} -\Delta \ell_{\text{max}}⪯B\left(u\left(k+1\right)-u\left(k\right)\right)⪯\Delta \ell_{\text{max}}, \end{array}$$ (7)

where *B* is the Jacobian matrix that can be considered as a constant matrix in each time cycle and is computed based on *x*(*k*) and *ℓ*(*k*).

To facilitate optimal target trajectory planning, the robot system is first discretized. Assume that the target position is unchanged with respect to the platform, *i.e*., (*r*_*gu*_, *ψ*_*gu*_) is constant, then (1) can be represented as *x* = *f*(*u*), where *x* and *u* are the 6DoF position vectors of the target and the platform, respectively. The discrete system is given by

$$\begin{array}{c} x(k)=f(u(k)). \end{array}$$ (8)

A general optimal target trajectory planning can be formulated as

$$\begin{array}{c} \begin{array}{cc} \text{minimize} & \sum_{k=0}^{\infty }h\left(x\left(k\right),\hat{x},u\left(k\right)\right),\\ \text{subject}\;\text{to} & (6),\;(7)\;\text{and}\;(8). \end{array} \end{array}$$ (9)

This nonlinear optimization problem can be approximated as a problem on linear systems. The linearization is performed in each time cycle, so it can describe the system in each small motion accurately. Consider *x*(*k* + 1) = *f*(*u*(*k* + 1)) and a small platform motion in each step *v*(*k*) = *u*(*k* + 1) − *u*(*k*). By linearization, the motion compensation discrete system is given by

$$\begin{array}{c} x(k+1)=x(k)+Av(k), \end{array}$$ (10)

where the matrix *A*

$$\begin{array}{c} A=\frac{\partial f}{\partial u}{|}_{r_{u},\psi_{u},r_{gu},\psi_{gu}\left(k\right)} \end{array}$$ (11)

depends on both (*r*_*u*_, *ψ*_*u*_) and (*r*_*gu*_, *ψ*_*gu*_) of moment *k*, and can be updated in each step.

The target can move back to the desired position following a straight-line in the target space, and such a trajectory can be obtained by defining an appropriate *h* in (9). This path is interpolated between *x*_*o*_ and $\hat{x}$,

$$\begin{array}{c} x\left(k\right)=x_{o}+\left(\hat{x}-x_{o}\right)\mu , \end{array}$$ (12)

where the scalar 0 ≤ *μ* ≤ 1 is *k* dependent, and *μ* can be maximized for a quick motion compensation to the desired target position $\hat{x}$. The optimization can be implemented step-by-step. For simplification, consider only mechanical constraints (7). In each step, let *x*_*o*_ = *x*(*k* − 1). The desired next target position is (12), and the required platform motion is $v=A^{-1}\left(\hat{x}-x_{o}\right)\mu$. The scalar *μ* should be found to minimize $h={\left(x(k)-\hat{x}\right)}^{⊤}(x(k)-\hat{x})={\left(\mu -1\right)}^{2}{\left(x_{o}-\hat{x}\right)}^{⊤}(x_{o}-\hat{x})$. This is equivalent to minimize (*μ* − 1)^2^, subject to $-v_{\text{max}}⪯A^{-1}\left(\hat{x}-x_{o}\right)\mu ⪯v_{\text{max}}$. Thus, it can be verified that the fastest straight target trajectory is given by

$$\begin{array}{c} \begin{array}{c} x\left(k+1\right)=x\left(k\right)+(\hat{x}-x\left(k\right))\mu ,\\ \;\mu =\text{min}(1,1/\text{max}|\left(A^{-1}\left(\hat{x}-x\left(k\right)\right)\right)/v_{\text{max}}|). \end{array} \end{array}$$ (13)

Refer to such target straight-line planning as *target-S*. In target-S, the target follows a straight line for the start point to the end point, and the required platform and actuator length follow curved lines in operational space and joint space, respectively (Fig 1).

As forcing the robot to move the target along a straight-line path can lead to over constraining the system, it does not fully exploit the robot’s potential in reducing the target’s position error in a temporally optimal way. To include both spatial and temporal components in the optimization, a steepest descent of target position error in each step can be used by formulating (9) as follows,

$$\begin{array}{c} \begin{array}{cc} \text{minimize} & {\left(x_{o}+Av-\hat{x}\right)}^{\text{T}}\left(x_{o}+Av-\hat{x}\right),\\ \text{subject}\;\text{to} & -v_{\text{max}}⪯v⪯v_{\text{max}}. \end{array} \end{array}$$ (14)

For each step, *x*_*o*_ is updated, *x*_*o*_ = *x*(*k* − 1), and according to (10), the next step target position is *x*(*k*) = *x*_*o*_ + *Av*, where the vector *v* = *u*(*k*) − *u*(*k* − 1) is the platform motion to be optimized. This fastest reduction in target error positional error planning (14) will be referred to as *target-D* planning.

### Evaluation of robotic compensation for standard motions

The robotic SRS system was evaluated by two synthetic motion standards: step-like motion and oscillating motion. Step motion was simulated since it can represent system response performance to a sudden target change. Sinusoidal motion was considered since it evaluates how well the system responds to dynamical motion and is closely related to target oscillation caused by respiratory motion.

### Evaluation of robotic compensation for volunteer head motion data

Both SRS and couch robots were tested using previously recorded human data. Real-time 6D head positional data was obtained under IRB approved studies IRB14-0040 and IRB14-0535 at the University of Chicago [22]. Real-time 6D prostate tumor motion was obtained from the publicly available ACRF Image X Institute patient motion repository Alnaghy et al [44].

## Results

### Step-like and sinusoidal target motion

**Fig 3:**
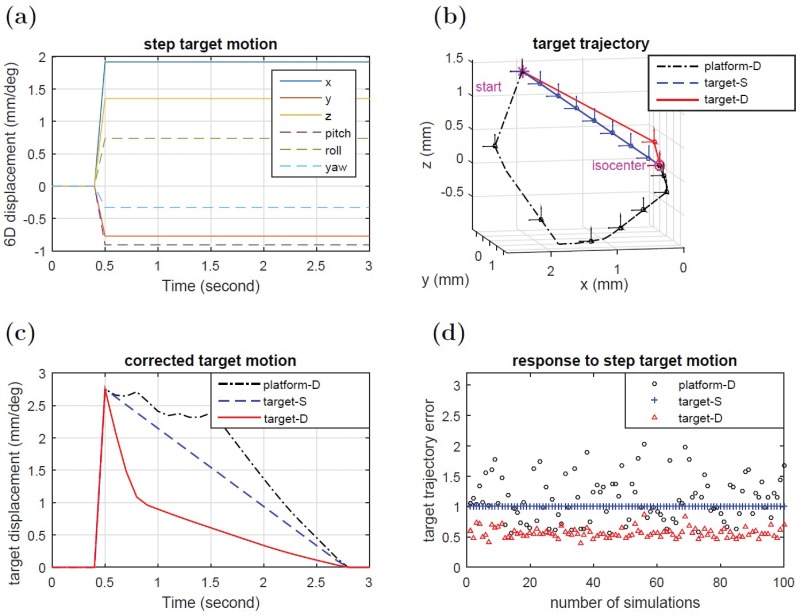
(a)(b)(c): System response to step like target deviations. (a) Input motion. (b) Target trajectory with orientation shown every 0.15 seconds. (c) Target displacement versus time. (d) Integrated trajectory errors for 100 simulations using randomly generated 6D input motion within 2mm/1deg. Errors were normalized to target-S planning case.

The simulated robotic SRS system was tested against sudden step-like displacements of the target away from the setpoint (Fig 3(a)). System response to this motion using platform-D control, target-S planning and target-D planning is shown in Fig 3(b). Here each point represents a 0.15 s time interval and the vector arrows attached to each point denote rotations. Although all planning strategies eventually converged to the setpoint, it can be seen that certain strategies were more appropriate to motion compensation in RT. For platform-D planning (black line), the target can deviate significantly from a straight-line path and can take a long time to converge. In this case, the target temporally moved further away and took approximately 1.6 s to reach a value less than 1 mm/deg away from the setpoint. From a target perspective, such a trajectory is not optimal as it would lead to unnecessary irradiation of healthy tissues. For target-S planning (blue line), the target moved at a constant speed and followed the expected straight-line path. However, although optimal spatially, this trajectory is not optimal temporally as it takes approximately 1.5 s to reach less than 1 mm/deg away from the setpoint. For target-D planning (red line), the target followed a path that was close to a straight-line and converged to the setpoint within 0.3 s. In this case, both spatial and temporal components of the trajectory were optimal. The time course $∥x\left(k\right)-\hat{x}∥$ of the three trajectory planning strategies is given in Fig 3(c).

To test system response against many arbitrary 6D directions, 100 randomly generated target step-like displacements with amplitudes within a 2mm/1deg were inputted into the simulated robotic SRS system. The performance of different control/planning methods was evaluated by the target trajectory error defined as

$$\begin{array}{c} E=\sum_{k=1}^{\infty }∥x\left(k\right)-\hat{x}∥, \end{array}$$ (15)

where the difference from the desired setpoint is summed over all time steps. The target trajectory errors of 100 simulations are plotted in Fig 3(d). The trajectory errors of target-S planning were smaller than those of platform-D control in 56 cases, and target-D planning had the smallest trajectory error in all 100 cases. On average, target-D planning was found to have a 45% smaller target trajectory error than platform-D control, and a 44% smaller target trajectory error than target-S planning.

**Fig 4:**
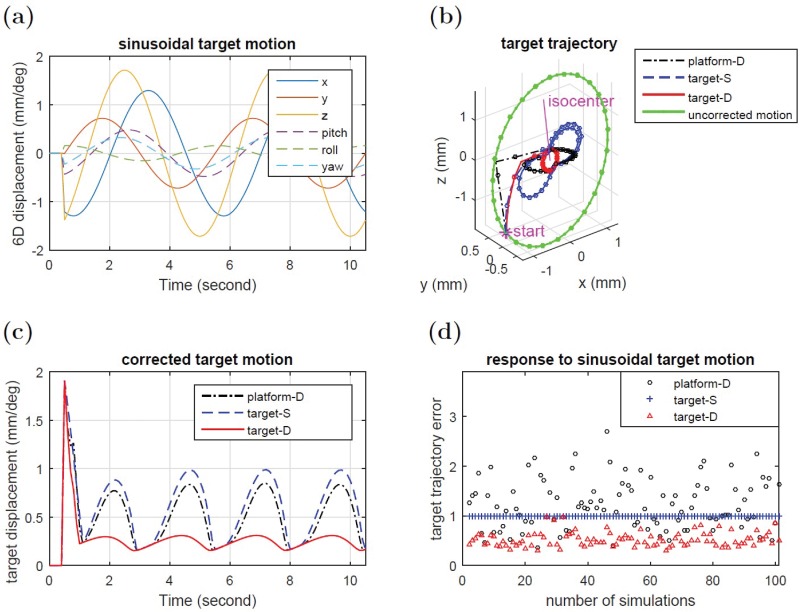
(a)(b)(c): System response to sinusoidal target motion deviation. (a) Input sinusoidal target motion. (b) Target trajectory shown every 0.2 seconds. (c) Target displacement versus time. (d) Integrated target trajectory errors for 100 simulations using randomly generated input motion within 2 mm / 1 deg. Errors were normalized to target-S planning case.

The performance of optimization based trajectory planning was also evaluated for sinusoidal target motion around the desired setpoint. Fundamentally, due to lag time between measurement and robot actuation, the corrected target position will not remain at the setpoint, but rather, the target will move around the setpoint with a smaller amplitude of oscillation. This amplitude can therefore be used as an indicator of the efficiency of the motion correction algorithm. The system response of platform-D control, target-S planning and target-D planning for a randomly generated 6D target oscillation is shown in Fig 4(a), 4(b) and 4(c). The trajectory of target-D planning was found to converge to a steady oscillation faster than platform-D control and target-S planning, and also showed a smaller oscillation amplitude. Fig 4(d) shows the resultant errors for 100 simulations using randomly generated sinusoidal input motion with amplitudes within 2mm/1deg. Target-S planning has smaller target trajectory errors than platform-D control in 68 cases, and target-D planning has the smallest target trajectory errors in all 100 cases. On average, target-D planning has 52% smaller target trajectory error than platform-D control, and has 48% smaller trajectory error than target-S planning.

### Human volunteer motion

**Fig 5:**
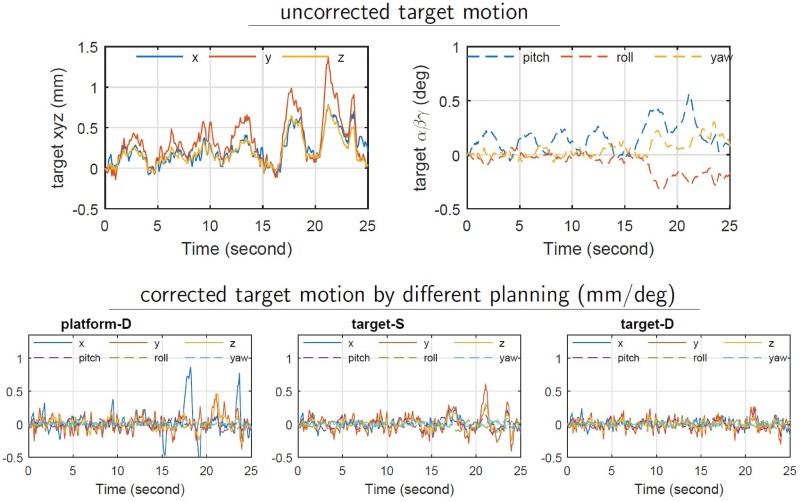
Different trajectory planning strategies for real-time motion compensation of volunteer head motion showing strong respiratory coupling.

Uncorrected 6DoF head motion of six volunteers was record over a 15 minute tracking period by use of a stereoscopic IR marker tracking system with a 12 Hz sampling rate [22]. In all cases no immobilization was applied to the volunteers. The motion was inputted into the robotic SRS simulation, and platform-D control, target-S planning, and target-D planning were applied, and their performances compared. Fig 5 is one example showing a volunteer displaying involuntary drifting and rapid head motion changes due to respiratory coupling. In his case platform-D control fails to meet the 0.5 mm / 0.2 deg tolerance objective, whereas, the target-D planning is well within tolerance.

**Fig 6:**
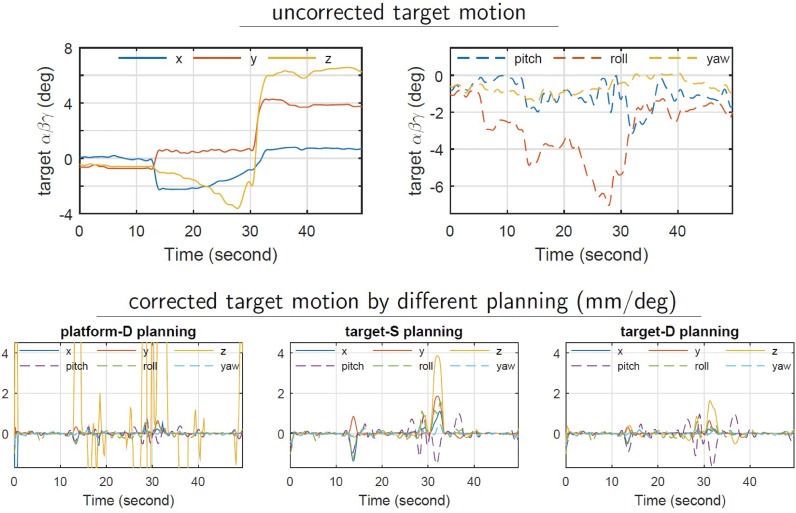
Different trajectory control/planning strategies for real-time motion compensation of a prostate tumor using a 6DoF robotic treatment table.

To demonstrate the algorithm’s applicability to many different types of 6DoF robot systems, Fig 6 shows the results of performing real-time 6D prostate motion compensation using a treatment table. If one were to use a platform-D control to move the platform to the required position as fast as possible, it leads to large intermediate target position errors. On the other hand, both target-S planning and target-D planning were acceptable, while the displacement for target-D planning is within tolerance 1.5 mm / 1.5 deg.

## Discussion

As the radiation beam is always on during the motion compensation process, the 6D correction trajectory must be optimal both spatially and temporally in order to maximize radiation to the target and minimize unintentional irradiation of healthy tissues. As can be seen in Fig 3(b) for a sudden step-like deviation, simply moving the patient support device to the desired position without regard to the target trajectory can result in highly curved trajectories. Although such trajectories are completely acceptable in the vast majority of robot applications, where one is only concerned with moving an object from point A to point B, this is not acceptable in real-time motion compensation using RT as such trajectories may temporally bring OARs, or other sensitive structures, directly into the path of the radiation beam. On the other hand, forcing the target to move along an ideal straight-line, or any well-defined 6D path in target space, can lead to prolonged correction times (target-S plan) as the system becomes over-constrained, in that the robot must move its joints in a fixed way. The use of optimization to explore all potential 6D trajectories by taking into account the robot’s joint configuration allows for the various degrees of freedom of the robot to be fully exploited. For example, target-D planning was found to provide a good balance between both spatial and temporal efficiency. As shown in Fig 3(c), the 6D target error is quickly reduced, such that in 0.3 s it is approximately 1 mm/deg away from isocenter, compared to ≈ 2s using the other approaches.

Typically, in lung tumor motion compensation, or for other targets coupled to respiratory motion, the high velocities necessitate the use of prediction algorithms in order to account for the inherent lag time between actual target position readout and the motion controller response. As shown in Fig 4, a less optimal correction trajectory will have larger lag times and consequently require more motion prediction. Numerous studies have shown, that the accuracy of prediction algorithms becomes substantially more difficult the further the algorithm must predict into the future [45]. In this respect, optimization based trajectory planning can improve the accuracy of such motion compensation systems in that an optimal 6D correction path would reduce lag time and consequently would require less prediction. Fig 4 compares several different trajectory planning strategies in handling 6D oscillatory motion. As shown in Fig 4(b), all three different planning strategies reach equilibriums that orbit around the setpoint. However, the target-D planning method comes closest to the desired setpoint, which is primarily due to its correction path being more optimal than the other methods.

The optimization based motion planning method presented in this work is general in nature and can be applied to any robotic system. The main difference between the compact hexapod and the 6DoF patient treatment table system investigated in this work is the joint space. By defining the table joint space, the method can be fully utilized in order to find optimal 6D trajectories. This is shown in Fig 6, where real-time motion compensation of prostate motion is performed using the treatment table. However, it should be noted that patient tables are typically designed for general purpose treatment in all parts of the body, and therefore typically have mechanical joint positions inferior of the patient’s pelvis in order to prevent significant attenuation or scattering of the radiation beam. When treating targets in the brain, these joints are located far from isocenter and using such a system for SRS motion compensation will pose high demands on the control system in terms of motor synchronization due to mechanical translational and rotational shift. For example, the treatment table investigated in this work (TruePitch, Varian Medical Systems, CA) [46], has a pitch/roll pivot point located approximately 1500 mm away from the patient’s head. Therefore, a small 0.05deg pitch error can cause a 1.31 mm displacement error in the vertical direction of the target. On the other hand, site specific robots such as the robotic SRS system are located near the patient’s head, and have a joint-to-isocenter distance that is approximately 1/4—1/3 that of a table, allowing for less demanding mechanical system tolerances.

In order to find the most optimal way to move the robot, the optimizer operates directly on the robot’s joint space. Therefore, this technique is more demanding to implement than other motion controllers, such as PID, which can treat the robot like a black box and adjusts inputs until a desired output is achieved. In this case complete knowledge of the robot’s joint position geometry together with dynamical capabilities of the motor systems is needed. Failure to provide accurate mechanical and dynamical robot constraints can lead to systematic errors where the optimizer finds trajectory solutions that result in target trajectory errors.

In optimal trajectory planning, it was assumed that we know the robot mechanical structure, actuator positions, and the necessary coordinate transformations to calibrate the robot to the LINAC frame of reference. In actual clinical practice, there many be robot flex, mechanical play, or other conditions that will lead to deviations from this ideal. In this case, it is necessary to perform a robot calibration procedure. For robotic SRS, such a calibration was demonstrated by pre-programming the robot to move to different positions in 6D space while simultaneously monitoring the actual positions with respect to the LINAC frame using a real-time 6D tracking system. A Kabsch algorithm was then used to compute coordinate frame relations between the robot and LINAC frames [34]. We are currently investigating this technique for use on 6DoF couch systems.

## Conclusion

A general 6D target trajectory optimization framework for robotic patient motion compensation systems was investigated. Motion planning was formulated as an optimization problem and solved in real-time using a well-known convex optimization algorithm. The method was found to be flexible as it meets various performance requirements such as mechanical robot limits, patient velocities, or other aspects of the system that must operate within fixed limits during the motion compensation process. Both straight-line path and steepest decent trajectories of target error were investigated. In all cases, the steepest decent of the target trajectory was optimal both spatially and temporally.
